# Procedures and timetable for proposals to amend Chapter F of the International Code of Nomenclature for algae, fungi, and plants

**DOI:** 10.3897/imafungus.17.195573

**Published:** 2026-05-07

**Authors:** Tom W. May, Konstanze Bensch

**Affiliations:** 1 Royal Botanic Gardens Victoria, Birdwood Avenue, Melbourne, Victoria 3004, Australia Fungal Nomenclature Bureau, XIII International Mycological Congress Incheon Republic of Korea; 2 Fungal Nomenclature Bureau, XIII International Mycological Congress, Incheon, Republic of Korea Royal Botanic Gardens Victoria Melbourne Australia; 3 Westerdijk Fungal Biodiversity Institute, Uppsalalaan 8, 3584 CT Utrecht, Netherlands Westerdijk Fungal Biodiversity Institute Utrecht Netherlands

**Keywords:** Fungal Nomenclature Bureau, Fungal Nomenclature Session, Governance, International Mycological Association, International Mycological Congress, Madrid Code

## Abstract

Guidelines for preparing and submitting proposals to modify or enhance Chapter F of the International Code of Nomenclature for algae, fungi, and plants are set out here. Such proposals will be considered by the Fungal Nomenclature Session of the XIII International Mycological Congress, to be held in Incheon, Korea in August 2027. A timetable is established for the submission of proposals, due by 31 December 2026, their publication in IMA Fungus, the release of the ‘Synopsis of proposals’ and the conduct of the pre-Congress guiding vote.

## Introduction

In the International Code of Nomenclature for algae, fungi, and plants (Code), the current edition of which is the Madrid Code (Turland et al. 2025), Chapter F assembles the provisions that are specific to fungi. The governance of material in Chapter F falls under the remit of the International Mycological Congress (IMC) (see Table 1). The Code stipulates (Division (Div.) III, Provision (Prov.) 1.2, 8.1) that Chapter F may be altered only by decision of a plenary session of an IMC on a resolution moved by the Fungal Nomenclature Session (FNS) of that Congress. The current Chapter F was incorporated directly into the Madrid Code, published on 25 July 2025 in print, eBook and PDF editions (Turland et al. 2025) and on 4 September 2025 in an open-access interactive online edition (https://www.iaptglobal.org/_functions/code/madrid). It includes modifications to the preceding version of Chapter F which was the San Juan Chapter F (May et al. 2019). The amendments were adopted on 15 August 2024 at IMC12 in Maastricht, the Netherlands.

**Table 1.** Key documents, organisations and events related to fungal nomenclature.

Documents, organisations and events related to fungal nomenclature
Chapter F — the section of the Code containing provisions pertaining solely to fungi
Editorial Committee for Fungi (ECF) — elected at the FNS, makes the necessary changes to Chapter F based on formal proposals accepted at a FNS
Fungal Nomenclature Bureau (FNB) — organises and runs the FNS at an IMC
Fungal Nomenclature Session (FNS) — held during each IMC; considers proposals to amend or enhance Chapter F
Guiding vote — a non-binding indication of opinions on the proposals to amend or enhance Chapter F; held prior to the FNS
International Code of Nomenclature for algae, fungi, and plants (Code)
International Mycological Association (IMA)
International Mycological Congress (IMC) — organised by the IMA; decisions of the FNS are ratified during the plenary session of the IMC
Nomenclature Committee for Fungi (NCF) — elected at the FNS, considers proposals to conserve or reject names of fungi; advises on, but does not decide on proposals to amend Chapter F

The next International Mycological Congress (IMC13) is scheduled to be held in Incheon, Korea in August 2027 with the Fungal Nomenclature Session taking place during the Congress. The procedures of the FNS are set out in Div. III Prov. 1, 2, 4, 5 and 8 of the Code and closely mirror those of the Nomenclature Section of an International Botanical Congress (IBC), with the exception that no institutional votes are utilised at an IMCFNS. The Fungal Nomenclature Bureau (FNB) of an International Mycological Congress is responsible for organising the FNS and the pre-Congress guiding vote. The FNB is composed of various members, including the Secretary and Deputy Secretary, who fulfils the role equivalent to the Rapporteur-général and Vice-rapporteur in the Bureau of Nomenclature of an IBC (May and Bensch 2024). Procedures for the appointment of FNB officers are established in Div. III Prov. 4 and 8. The Secretary for each IMC is elected at the preceding IMC. At IMC12 in Maastricht, Tom W. May was re-elected Secretary of the FNB for IMC13 (May et al. 2024). The Deputy Secretary of the FNS is “appointed by the Secretary and approved by the Nomenclature Committee for Fungi [NCF] in consultation with the General Committee …” (Div. III Prov. 8.7). In a ballot of the NCF in March 2025, Konstanze Bensch was confirmed as Deputy Secretary of the Fungal Nomenclature Bureau for IMC13 on the suggestion of the Secretary and after consultation with the General Committee.

## Procedure for amending Chapter F of the Code

The procedure for proposing modifications to Chapter F of the Code is as follows:

- After each new version of Chapter F appears, proposals for amendment are published in IMA Fungus, either singly or bundled together (see below). Such proposals may suggest revised wording for existing Articles or Recommendations of Chapter F and/or introduce new material dealing solely with names of organisms treated as fungi (including fossil fungi).
- Prior to the next International Mycological Congress, a ‘Synopsis of proposals’ assembles all published proposals to amend Chapter F, arranged by Article and Recommendation and re-publishes them in IMA Fungus. The synopsis omits the justification that accompanied the original publication, but is a commentary from the Secretary and Deputy Secretary of the FNB from a technical perspective — with particular attention to the clarity of wording, implications for other articles and any unintended consequences. The synopsis also includes the opinions of relevant bodies, such as the Nomenclature Committee for Fungi (NCF).
- A pre-Congress guiding vote is held online, commencing shortly after the Synopsis of proposals is published. The guiding vote is a non-binding indication of the views of those entitled to participate, as set out in Div. III Prov. 8.3 — principally members of the International Mycological Association (IMA), authors of proposals and members of the NCF. Persons eligible to vote will be contacted, either directly by the Secretary of the FNB or through the IMA or relevant societies. The outcomes of the guiding vote are made available on the IMA website in advance of the FNS of the Congress.
- The Fungal Nomenclature Session, convening during the International Mycological Congress, considers the published proposals, including any amendments to them. For each proposal, a formal vote amongst those individuals present on the day is taken.
- Decisions of the FNS are ratified by vote of the plenary session of the Congress. At that point, the amended provisions take effect.
- The new version of Chapter F, incorporating the changes accepted at the Congress, is prepared by the Editorial Committee for Fungi (elected at the FNS), in consultation with the Editorial Committee for the Code as a whole.
- The new edition of Chapter F is published. The online version of the Code is amended with a note pointing to the new edition of Chapter F.

## Timetable and rules for proposals

In our capacities as Secretaries of the Fungal Nomenclature Bureau and editors of proposals to amend Chapter F, we have decided that the following rules should apply to proposals submitted for consideration by the Fungal Nomenclature Session of the XIII International Mycological Congress in Incheon (see Table 2). These rules largely follow those set out for proposals to amend the Code to be considered at IBC XXI in 2029 (Turland et al. 2026).

**Table 2.** Key dates for proposals to amend Chapter F of the International Code of Nomenclature for algae, fungi, and plants.

Stage in submission and processing of proposals	Date
Deadline for submission of proposals	31 December 2026
Publication of “Synopsis of proposals”	March 2027
Guiding vote	May 2027
Results of guiding vote	June 2027
Fungal Nomenclature Session (FNS) at IMC13	August 2027
Publication of revised “Incheon Chapter F”	Early 2028

- Proposals that fail to follow these instructions may be rejected for that reason.
- Submission of proposals is now open.
- The closing date for the submission of proposals is 31 December 2026.
- Proposals may be subject to external review.
- Late submissions received by 31 January 2027 may be accepted if they require neither review nor substantial editing.
- Proposals are to be submitted by e-mail to the Secretaries of the FNB (tom.may@rbg.vic.gov.au and k.bensch@mycobank.org).
- After editing, proposals will be made available on the IMA website ( http://www.ima-mycology.org/nomenclature/formal-proposals) and published in IMA Fungus under the Nomenclature category, with each proposal assigned a sequential number.
- We note that there is no article-processing charge for items accepted for publication in the Nomenclature category of IMA Fungus.
- Depending on the number of proposals, their length and the time of submission, individual proposals and their accompanying justifications may be published separately or grouped together for publication, as was the case prior to the preceding IMC (May and Hawksworth 2024).
- Proposals to amend Chapter F at IMC13 in 2027 must be based on the text of Chapter F of the Madrid Code (Turland et al. 2025).
- Each proposal should include an initial summarising paragraph.
- There should be an assessment of the impact of the proposal to the stability of nomenclature along with some general explanation about the rationale for the proposal.
- Proposals must be presented concisely and may be condensed by the Secretaries and/or the journal editors.
- Proposals to amend Chapter F submitted prior to the Maastricht IMC and published in IMA Fungus serve as examples (May and Hawksworth 2024).
- If a proposal results from a more general context, authors must make that context the subject of a separate paper to be submitted to IMA Fungus (or another journal), reviewed in the normal way and considered for acceptance in competition with papers on other topics. Such manuscripts are to be submitted via the IMA Fungus online manuscript submission system ( https://imafungus.pensoft.net). The associated proposal, including a cross-reference to the relevant paper, should be as concise as necessary and is to be submitted separately by e-mail to the Secretary of the FNB.
- In the case of a Special-purpose Committee, a report summarising the Committee’s work should be submitted to IMA Fungus (in the Nomenclature category), providing context and justification for any associated proposals, which are to be prepared and submitted as described above. One Special-purpose Committee has been established to report to the XIII International Mycological Congress: the Special-purpose Committee on Genomes as Types for Fungi, with Marco Thines as Convenor and Nathan Schoutteten as Secretary.
- Proposals should have a title that states the aim of the proposals, for example, “Proposals to permit naming of organisms known only from DNA sequences”, not merely referring to the numbering of the Code. Thus, do not use titles in the form: “Proposals to amend Article F.8.1 and F.10”.
- Each proposal must state explicitly what amendment is proposed to Chapter F, for example, “Insert a new Article after Art. F.6”; “Delete Art. F.10”; or “Amend Rec. F.5A.1 to read as follows (new text in bold, deleted text in strikethrough)”.
- Proposals must not consist simply of new text or explanation, but should indicate precisely what text is to be inserted, deleted or changed in Chapter F. This can be achieved by copying and pasting the text from the current version of Chapter F in the online edition of the Madrid Code and then showing new text in bold.
- A clearly worded, concise proposal is more likely to be read sympathetically than a long, complex argument or a series of repetitive proposals.
- If a general principle applies to several Articles and Recommendations, the matter can often be covered by a single proposal. If, for example, a proposal were made to alter the manner in which sanctioning is indicated, it would not be necessary to submit separate formal proposals for every single passage where sanctioned names appear.
- If acceptance of a proposal would require renumbering or rewording of the remainder of an Article or Recommendation, such changes would automatically be made by the Editorial Committee for Fungi and no separate proposals for these changes are required.
- Authors will be asked to revise or withdraw proposals that conflict with other parts of the Code or that would cause significant nomenclatural disruption.
- The Editorial Committee for Fungi (Div. III Prov. 7.13) (in consultation with the Editorial Committee) can insert, delete or change Examples and Glossary entries without a formal proposal (Div. III Prov. 7.12). Accordingly, proposals to insert, delete or change Examples in Chapter F or Glossary entries specific to provisions concerning fungi need not be published in IMA Fungus unless they are essential for the understanding of a proposal to amend an Article, Note or Recommendation. Instead, they may be submitted at any time directly to the Editorial Committee for Fungi (for matters relating to fungi) which, prior to the Incheon Mycological Congress, is represented by the Secretaries of the Fungal Nomenclature Bureau.

## Recommendations for proposals

- In general, proposals that contribute to nomenclatural stability are more likely to be successful.
- Proposals that reduce the complexity of the Code without negatively affecting nomenclatural stability are also likely to succeed.
- Proposals addressing nomenclatural situations that occur only rarely are unlikely to succeed, particularly if they add to the complexity of Chapter F. Such situations may be better addressed through the conservation or rejection of a particular name, suppression of a particular work, a request for a binding decision on a particular matter (as possible through Art. 38.5 or Art. 53.4) or a new Example in Chapter F.
- Authors are encouraged to provide a list of all the provisions of the Chapter F (Articles, Notes, Recommendations and Examples) believed to be affected by a given proposal. This helps the Editorial Committee for Fungi, whose main job is to ensure that any amendments adopted for the new Chapter F are fully integrated with the existing text and consistently implemented throughout.
- If Examples are included, they must follow the format used in Chapter F of the Madrid Code, i.e. with places of publication cited in full. Authors should provide links to online versions of those publications and the protologues of any basionyms, replaced synonyms or names causing illegitimacy. Online resources include the Biodiversity Heritage Library ( https://www.biodiversitylibrary.org), Biblioteca digital del Real Jardín Botánico CSIC ( https://bibdigital.rjb.csic.es), Gallica ( https://gallica.bnf.fr/), Google Books ( https://books.google.com/), HathiTrust ( https://www.hathitrust.org/) and similar collections. For publications not yet available online, a scanned copy of the relevant pages (including title pages) is requested.

## Proposals relevant also to other organisms

Some proposals concerning fungi may be equally applicable to all organisms covered by the Code or to taxonomic groups broader than fungi alone. If such proposals are submitted for consideration by the FNS of IMC13, they should be formulated so as to be restricted to fungi. Proposals to amend the Code that are not specific to fungi should be submitted to the journal Taxon in accordance with the instructions provided by Turland et al. (2026) by 31 Mar 2028, for consideration by the Nomenclature Section of the XXI IBC.

## Additional information

### Artificial Intelligence (AI) use

The authors accept full responsibility for the content of the manuscript, including the disclosure of any use of AI.

No AI tools were used in the preparation of this manuscript.

### Author ORCIDs

T.W. May https://orcid.org/0000-0003-2214-4972

K. Bensch https://orcid.org/0009-0006-3404-9955

### Data availability

All of the data that support the findings of this study are available in the main text.
